# Supplementary figures and images for: Mutation severity spectrum of rare alleles in the human genome is predictive of disease type
Source: PLoS Comput Biol. 2020 May 15;16(5):e1007775. doi: 10.1371/journal.pcbi.1007775 (PMC7255613; doi:10.1371/journal.pcbi.1007775)

**Supplemental Figure S1. DeepSAV neural network structure.**


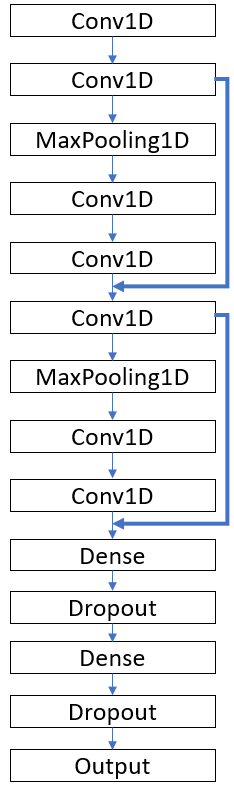

Supplement: S1 Fig — (DOCX) [file pcbi.1007775.s001.docx]
