# Supplementary material for: Mutation severity spectrum of rare alleles in the human genome is predictive of disease type
Source: PLoS Comput Biol. 2020 May 15;16(5):e1007775. doi: 10.1371/journal.pcbi.1007775 (PMC7255613; doi:10.1371/journal.pcbi.1007775)

**Supplemental Figure S2. ClistVis heatmap of potential disease-causing genes (UniProt label on the right) and genes with pathogenic SAVs (UniProt label on right with _P).** Scores (labeled below) for each gene are colored from blue (low) to red (high) and clustered (20 clusters delimited by spaces) according to complete linkage of correlation distances. Two clusters with low GTS scores (mutation intolerant) have a relatively high proportion of genes with known pathogenic variants and could help identify new disease-associated genes (red labels).


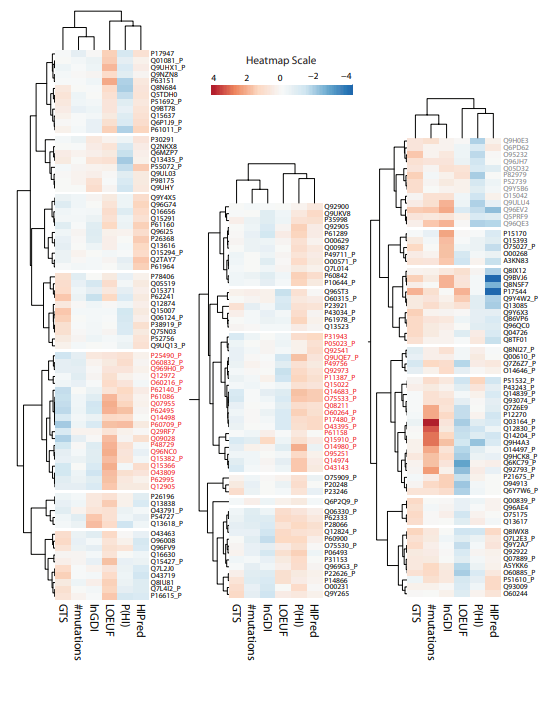

Supplement: S2 Fig — Scores (labeled below) for each gene are colored from blue (low) to red (high) and clustered (20 clusters delimited by spaces) according to complete linkage of correlation distances. Two clusters with low GTS scores (mutation resistant) have a relatively high proportion of genes with known pathogenic variants and could help identify new disease-associated genes (red labels). (DOCX) [file pcbi.1007775.s002.docx]
