# Supplementary material for: Mutation severity spectrum of rare alleles in the human genome is predictive of disease type
Source: PLoS Comput Biol. 2020 May 15;16(5):e1007775. doi: 10.1371/journal.pcbi.1007775 (PMC7255613; doi:10.1371/journal.pcbi.1007775)

**Supplemental Figure S3. The effects of feature combination and leave-out on neural network performance.** ROC AUC values are reported for neural network predictions made by using subsets of features. The notations of the predictors using subsets of features are as follows: AA – using only amino acid types; AA+seg: using amino acid types and low complexity region predictions; AA+coiled.coil: using amino acid types and coiled coil region predictions; using AA+sec.struct: amino acid types and secondary structure predictions; AA+uniprotFeat: using amino acid types and features derived from UniProt Feature fields; AA+disorder: using amino acid types and disorder propensity ; AA+consv: using amino acid types and sequence conservation; AA+prof: using amino acid types and sequence profile; AA+consv+prof: using amino acid types, sequence conservation, and sequence profile; ALL-prof: using all features except sequence profile; ALL-consv: using all features except sequence conservation; ALL-prof-consv: using all features except sequence profile and sequence conservation; ALL-uniprotFeat: using all features except those derived from UniProt Feature fields; ALL-disorder: using all features except disordered region predictions; ALL-coiled.coil: using all features except coiled coil region predictions; ALL-sec.struct: using all features except secondary structure predictions; ALL-seg: using all features except low complexity region predictions by seg; ALL: using all features.


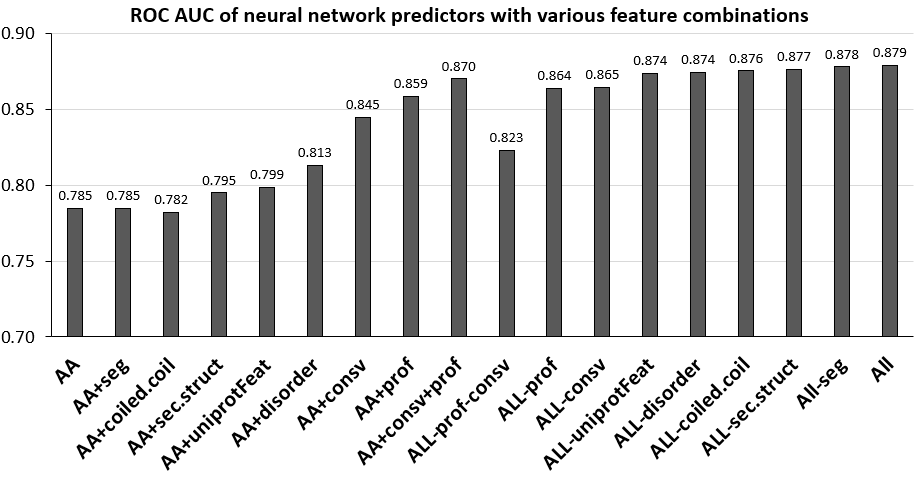

Supplement: S3 Fig — ROC AUC values are reported for neural network predictions made by using subsets of features. The notations of the predictors using subsets of features are as follows: AA–using only amino acid types; AA+seg: using amino acid types and low complexity region predictions; AA+coiled.coil: using amino acid types and coiled coil region predictions; using AA+sec.struct: amino acid types and secondary structure predictions; AA+uniprotFeat: using amino acid types and features derived from UniProt Feature fields; AA+disorder: using amino acid types and disorder propensity; AA+consv: using amino acid types and sequence conservation; AA+prof: using amino acid types and sequence profile; AA+consv+prof: using amino acid types, sequence conservation, and sequence profile; ALL-prof: using all features except sequence profile; ALL-consv: using all features except sequence conservation; ALL-prof-consv: using all features except sequence profile and sequence conservation; ALL-uniprotFeat: using all features except those derived from UniProt Feature fields; ALL-disorder: using all features except disordered region predictions; ALL-coiled.coil: using all features except coiled coil region predictions; ALL-sec.struct: using all features except secondary structure predictions; ALL-seg: using all features except low complexity region predictions by seg; ALL: using all features. (DOCX) [file pcbi.1007775.s003.docx]
